# Predictors of overload in parents of children with neuromuscular diseases

**DOI:** 10.3389/fneur.2024.1349501

**Published:** 2024-02-23

**Authors:** A. A. Rodríguez, M. García, Oscar Martínez, J. F. López-Paz, I. García, P. Pérez-Nuñez, I. Amayra

**Affiliations:** Neuro-e-Motion Research Team, Faculty of Health Sciences, Department of Psychology, University of Deusto, Bilbao, Spain

**Keywords:** parents, caregivers, neuromuscular diseases, overload, life satisfaction, somatic symptomatology

## Abstract

**Introduction:**

Parents of children with neuromuscular diseases experience multiple difficulties in their daily lives that affect their physical and psychological health. The risk factors for these health issues have not been sufficiently investigated. Therefore, the aim of this study was to analyze the potential predictors of overload in these parents, including QoL, somatic symptomatology, life satisfaction, psychological adjustment and certain sociodemographic variables.

**Methods:**

A cross-sectional research study was conducted among parents who are caregivers for children with NMD in Spain. A convenience sample of 110 parents who were contacted by associations and hospitals was used. Variables were evaluated using the sociodemographic questionnaire, CarerQol-7D, PHQ-15, Barthel Index, Psychological Adaptation Scale, Zarit Overload Scale and Satisfaction with Life Scale.

**Results:**

One of the most relevant findings of the present study is the identification of 3 overload groups (mild to moderate, moderate to severe, and severe overload) based on life satisfaction and somatic symptom scores within the predictive model of the discriminate analysis. Wilk’s lambda of the discriminant function was 0.568, χ^2^ (2, *n* = 55) = 8.815, *p* < 0.001.

**Discussion:**

This study presents a model that reveals the influence of unemployment, having a child with a severe level of dependency, the presence of somatic symptomatology and life satisfaction on caregiver overload. Likewise, the caregiver’s self-esteem could be a protective factor against overload.

## 1 Background

Paediatric neuromuscular diseases (NMDs) include a variety of rare disorders that are characterized by progressive muscle degeneration and muscle weakness, which lead to functional disabilities (1, 2). There are approximately 600 different NMDs affecting 1 in 3,000 individuals around the world (3). Most NMDs are genetic and manifest clinically during childhood (3). All NMDs have in common the following symptoms that eventually lead to dependence in the patient: muscle weakness, musculoskeletal deformity and pain, deteriorating respiratory and cardiac function loss of ambulation, and fatigue (4, 5). This results in a need for constant supervision and attention, thus most patients with NMDs live at home and receive daily assistance from family members, and the majority of them are usually parents. They become the main informal caregiver (6).

### 1.1 Caring difficulties associated to children with NMD

Caring for these patients requires close, continuous third-party attention, which can seriously affect their quality of life (QoL) (7, 8). In addition to the severity of the disease and symptoms, there are other comorbidities that are present during the child’s development that parents have to address, such as behavioral and emotional disturbances, deficits in social communication and adaptation, and cognitive symptoms (9).

Another problem is the continuous attendance at health centers and various therapies, which means that parents or other informal caregivers have to remember appointments and have reduced time for themselves (10). This is coupled with the difficulty that many parents have in accessing appropriate health services for their child (11).

As a result of these factors, chronic stress is one of the main challenges among informal caregivers (7, 8). Parents of children with NMDs have generally poorer mental health than those who do not have children with NMDs and are at a higher risk of developing mental health problems, such as anxiety and depression (4, 10, 12). Therefore, these parents may suffer from caregiver overload or burnout syndrome. Maslach and Jackson (13) first conceptualized burnout as a syndrome occurring frequently in workers with emotional fatigue and/or exhaustion as a main feature. As their emotional resources are depleted, workers become unable to give adequate effort (13). Pearlin et al. (14) and Zarit (15) explored the concept of caregiver overload. These studies highlight that it is important to think of overload not as a unitary event or phenomenon, but as a set of circumstances, experiences, responses, and resources that vary considerably among people with different impacts on a worker’s health and behavior.

### 1.2 Factors that influence caregiver overload

According to the theoretical models, not everyone suffers the same level of overload; there are certain variables that can determine whether a disease has a negative impact on one’s life (6). In NMDs, stress is influenced by the unpredictable course of the disease, social stigma, parental guilt about genetic transmission, the intrusion of extended family into their lives and role changes between parents (16, 17). Older age of the patient and the family member, as well as an advanced stage of the disease, are some of the sociodemographic and clinical variables that have also been found to be associated with caregiver overload (18, 19). Family circumstances, such as not having a partner or having more children in the family unit, can also be considered predictors of overload (20).

Other variables that have been found to increase the overload of these relatives are somatic symptoms. If an individual begins to develop somatic symptoms resulting from caretaking, there will be a high probability of suffering from this overload at some point (21). Another study found a higher degree of physical and emotional fatigue due to the work of “caring” for the dependent person is associated with a higher presence of cardiovascular somatic symptoms (22). It has been reported that parents of children with NMDs who had developed health problems (sleep problems, back problems and hypertension) were worried about not being as able to care for their child, which ultimately increased overload (4). Burnout syndrome can therefore be significantly influenced by individuals’ cognitions and how they perceive themselves, i.e., whether they perceive themselves as being able to cope with caregiving tasks. Ultimately, this can improve or worsen psychological adjustment to illness, a variable that has also been found to be a predictor of caregiving overload (23), becoming more relevant as the disease progresses (24). One of the factors within psychological adjustment is self-esteem, a variable that seems to positively influence the family’s coping with childhood illness (25).

In general, the stress and overload that parents may feel can lead to a decrease in their QoL (26, 27). The overload experienced by parents of children with NMDs has been found to be significantly associated with a negative perception of their own physical health, mood alterations, and diminished social life (28). Other studies with other chronic diseases have also found that caregivers’ overload reduces their QoL (29). Furthermore, QoL-related factors such as satisfaction with life may be negatively correlated with the severity of caregiver overload (30).

### 1.3 Justification for the study

Caregiver overload is considered a construct influenced by several factors, therefore, a better understanding of these factors may provide useful information for the appropriate development of support programs (20). Research and clinical practice show that optimal family well-being facilitates patients’ adjustment to the disease, improves their participation in treatment programs, and has a positive effect on clinical response to treatment (6).

Several studies have tried to identify those factors that may be related to caregiver overload to conduct prevention programs to avoid the psychological problems derived from it. However, to date, most studies analyzing the factors influencing overload in caregivers of children with NMD have focused on specific pathologies (6, 16, 19, 27, 31–33). Some authors have focused on assessing the influence of health status on caregiver overload in children with NMDs. Nevertheless, due to rarity of NMDs, these studies had smaller samples (13, 34–37). Other researchers have focused on descriptively analyzing the physical and emotional situation of the caregiver (38, 39). Finally, studies that have investigated the variables that influence overload have been conducted in very homogeneous samples (12, 17, 18). In addition, there are studies with heterogeneous diagnoses and with a large sample, but they do not analyze the influence of sociodemographic variables on overload (40). All these studies are of great scientific relevance and all of them have contributed to the improvement of the lives of people with these diseases. The present study aims to address the gaps found in the research.

Therefore, the main objective was to investigate factors that may be associated with overload for parents of children with NMDs in a more heterogeneous and representative sample and introduce variables that may influence caregiver overload, such as demographic variables of patients and caregivers, level of dependency of the child, somatic symptomatology, QoL, life satisfaction and psychological adjustment to the disease. Specifically, we hypothesized that low life satisfaction, low QoL, low scores on scales related to psychological adaptation, as well as high scores for somatic symptomatology and child disability, will predict the presence of overload. On the other hand, sociodemographic variables such as sex, age, marital status, occupation, educational level and type of neuromuscular disease will influence overload.

## 2 Materials and methods

### 2.1 Participants

A cross-sectional research study was conducted among parents who are caregivers for children with NMD in Spain. The study involved a sample of 110 parents who were recruited from multiple sources, including NMD associations like ASEM and BENE, as well as Basurto Hospital and the University Hospital of Cruces. The participants in this study were selected based on specific criteria:

Inclusion criteria: (a) be a parent of a child diagnosed with an NMD; (b) be at least 18 years old; (c) be willing to provide the informed consent before participating in the study; (d) be a resident in Spain and have Spanish as one of their primary languages of communication; and (e) being the primary caregiver for children under the age of 18.

Exclusion criteria: (a) any other diagnosis unrelated to an NMD; (b) any other psychological or psychiatric diagnosis not related to the diagnosis of NMD; (c) unaddressed sensory impairments that hinder the proper execution of the evaluation protocol; and (d) illiteracy and not having access to a computer.

### 2.2 Measures

#### 2.2.1 Sociodemographic data

Sociodemographic data was collected by a 17-item *ad hoc* questionnaire, which collected the participants’ sociodemographic data (e.g., sex, age, academic level, type of employment and marital status).

#### 2.2.2 Somatic symptomatology

The PHQ-15, developed by Kroenke et al. (41), was employed for evaluating the somatic symptoms linked to caregiving, in its Spanish version (42). This questionnaire comprises 15 items designed to gauge various physical issues that caregivers might have experienced over the past four weeks. Research has shown strong internal consistency, with a Cronbach’s alpha coefficient of 0.78 (42). In this study, the Cronbach was 0.88.

#### 2.2.3 Caregiver overload

The Zarit Caregiver Overload Scale, developed by Zarit et al. (43), was employed to evaluate the sense of overload experienced by participants because of their caregiving responsibilities. The scale comprises 22 items that are rated on a five-point Likert-type response scale. Previous research, such as the study conducted by Ramírez et al. (44), has demonstrated strong internal consistency for this scale, with a Cronbach’s alpha coefficient of 0.91. In our current study, the Cronbach alpha coefficient yielded a similarly high value of 0.90.

#### 2.2.4 Satisfaction with life

The Satisfaction with Life Scale (SWLS) (37) was used to evaluate the life satisfaction of caregivers, throughout 5 questions. Each question is rated on a 5-point scale, where 1 means “strongly disagree,” and 5 signifies “strongly agree.” This results in total scores ranging from 5 to 25. In contrast, other international versions typically use a Likert-type scale ranging from 1 to 7. The concept of life satisfaction entails a subjective cognitive process in which individuals evaluate their overall satisfaction with their present life circumstances concerning self-defined standards or expectations of their ideal life (45). Previous research has indicated strong internal consistency for this tool, with a Cronbach’s alpha coefficient of 0.89, as demonstrated by Sarid et al. (46). The Spanish version of the SWLS has also displayed robust internal consistency, with Cronbach’s alpha coefficients ranging from 0.79 to 0.89, as reported by Atienza et al. (47). In this study, the Cronbach alpha coefficient was 0.88.

#### 2.2.5 Quality of life

QoL was evaluated using the CarerQol questionnaire developed by Hoefman et al. (48). This questionnaire focuses on measuring the QoL specifically related to caregiving and comprises 7 items. The Intraclass Correlation Coefficients (ICCs) for CarerQol-7D ranged from 0.55 to 0.94 in their study (49). The well-being aspect, assessed as happiness, was measured using the Visual Analog Scale (VAS) in the CarerQol-VAS, with the scale endpoints ranging from “completely unhappy” to “completely happy”. Subjective overload, as evaluated by the CarerQol-7D, was measured across seven dimensions: self-realization, relationship with the patient, mental health, economic problems, activities of daily living, external support, and physical health (50). The internal consistency of the CarerQol-7D has been assessed in previous studies, with Cronbach’s alpha coefficients of 0.64 (51) and 0.62 (52) reported. In our current study, the Cronbach alpha coefficient was determined to be 0.63.

To calculate a CarerQol-7D utility score based on the responses to these seven dimensions, utility tariffs for the CarerQol were developed. This score ranges from 0 (“worst imaginable caregiving situation”) to 100 (“best imaginable caregiving situation”), and its computation involved the use of discrete choice experiments. Version 1.1 of the CarerQol-Tariff, developed by Voormolen et al. (53), was employed in this study.

#### 2.2.6 Level of functional independence

The Barthel Index, developed by Mahoney and Barthel (54), was employed to evaluate the degree of functional independence in personal activities of daily living. This assessment tool has demonstrated strong internal consistency in prior research, with reported values ranging between 0.86 and 0.92, as indicated by Cid-Ruzafa and Damián-Moreno (55). In the present study, the Cronbach alpha coefficient yielded a similarly high level of internal consistency, with a value of 0.87. This assessment was administered to the caregivers as part of our research protocol.

#### 2.2.7 Psychological adjustment

The Psychological Adaptation Scale (PAS) was administered to assess adaptation to a chronic condition or risk of disease. It has four factors: coping efficacy, self-esteem, social integration, and spiritual meaning (56). A Cronbach alpha coefficient of 0.91 was obtained for the Spanish adaptation (23). In the current study, the Cronbach alpha coefficient was 0.94.

### 2.3 Procedure

The associations contacted the parents of affected children who were their primary caregivers, and those who were interested in taking part in the study received information regarding the evaluation process. This information was sent through an infographic detailing the information of the study and a presentation letter. If they agreed to participate, they contacted the association and were sent an email with the link to the survey. Data collection was carried out through a self-administered protocol accessible via a personal link on the “Qualtrics” virtual platform and all caregivers provided their informed consent. The duration of the protocol was approximately 1 h. Additionally, a telephone number was provided to them, allowing them to contact the researcher for any clarifications or questions that might arise during the survey completion process. This research received approval from the Responsible Ethics Commission (Ref: ETK-39/18-19) and was conducted in adherence to the principles outlined in the Declaration of Helsinki.

### 2.4 Statistical analysis

**Figure 1:**
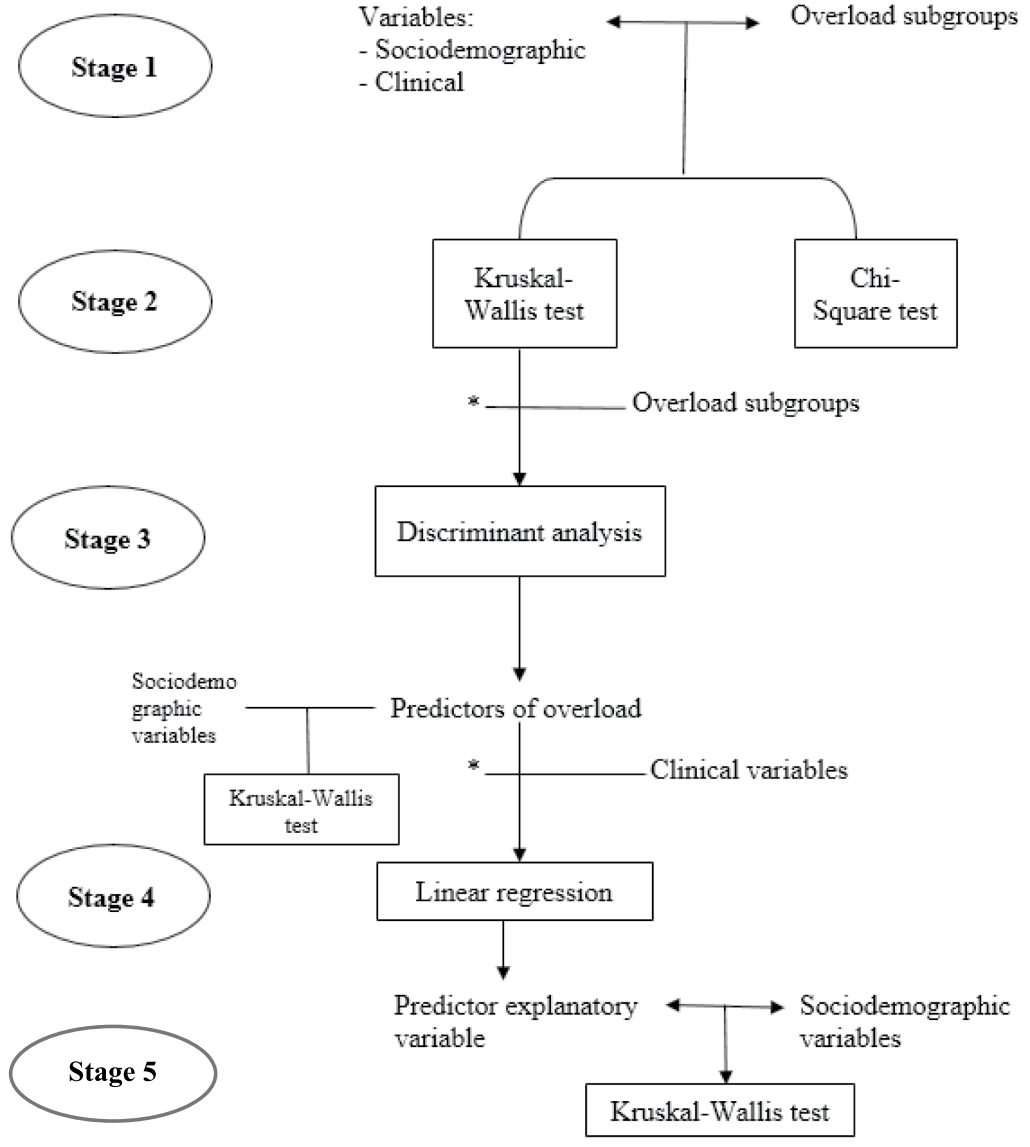
Stages of statistical analysis. *Those that were statistically significant in the previous section were considered for the subsequent analyses.

Descriptive measures were used to analyze the demographic data and clinical variables. Continuous variables are described by the mean and standard deviation, and categorical variables are described by the frequency and percentage. The current study involved five stages of analysis, all of which were conducted using SPSS, version 13.0. The 5 stages can be seen graphically in Figure 1.

The first stage involved the creation of four overload subgroups based on caregivers’ patterns of overload scores. To analyze whether there is a model that explains the presence of caregiver overload, the cut-off points used in this study were the internationally used cut-off points indicated by the authors of the instrument. Scores below 20 indicate little or no overload, scores between 21 and 40 indicate mild to moderate overload, scores between 41 and 60 indicate moderate to severe overload, and scores between 61 and 88 indicate severe overload (15). Because the last group of severe overload only included 5 people, this group was eliminated because it was not considered representative for the analyses. Thus, the mild group consisted of 20 participants; the mild to moderate group consisted of 37; and the moderate to severe group consisted of 34.

The second stage of analysis comprised the Kruskal–Wallis and Chi-square contrast tests for a preliminary inspection of the differences between groups in sociodemographic and clinical variables. The relationship between categorical variables was assessed by Chi-square analysis and the relationship between categorical and continuous variables by Kruskal-Wallis.

The third stage consisted of performing discriminant function analysis with the overload groups as the outcome variable and the significant variables between the previous analysis, in order to know the overload predictors. In addition, a Kruskal-Wallis analysis was performed to determine which socio-demographic variables influence these predictors of overload.

The fourth stage included a linear regression. Before carrying out linear regression, a Spearman’s correlation was conducted. Linear regression was carried out to determine to what extent somatic symptoms and life satisfaction were explained by other clinical variables. For the stepwise multiple linear regression, “model fit” was selected; the probability of using F was set at 0.05, and the constant was included in the equation. For the multiple linear regression, all standard and raw scores were converted into z scores. Variables with a high correlation were excluded from this model.

The fifth stage included a Kruskal-Wallis test to identify factors that may influence the explicative variables of predictors.

## 3 Results

### 3.1 First stage

A convenience sample of 110 people out of 200 who were contacted by associations and hospitals was used, ensuring that these were the primary caregivers of the affected children. The participants included 91 women (average age was 44.67 ± 7.25) and 19 men (average age was 47.42 ± 11.07). Geographical distribution was representative of all the autonomous communities that conform the Spanish state. Table 1 shows the data related to the sample distribution according to the type of NMD, marital status, educational level, and employment status. Table 2 shows descriptive measures related to demographic data, described by the mean and standard deviation.

**Table 1:** Sample distribution according to various sociodemographic variables in caregivers of children with NMD.

Variable		Frequency	Percentage
Gender	Male	19	17.2
	Female	91	82.8
Marital status	Married	84	76.4
	Cohabitating with a partner	7	6.4
	Divorced	8	7.3
	Separated	5	4.5
	Single	6	5.4
Education level	No school certificate	4	3.63
	Compulsory education	11	10
	Post-compulsory education	15	13.63
	Vocational education (intermediate)	12	10.9
	Vocational education (higher)	16	14.54
	Intermediate degree	16	14.54
	Bachelor’s degree	23	20.9
	Graduate	5	4.55
	Master’s degree	2	1.81
	PhD	6	5.45
Employment status	Employed	57	51.81
	Unpaid work	3	2.72
	Self-employed	7	6.36
	Unemployed	17	15.45
	Retired	5	4.54
	Housework	12	10.9
	Student	7	6.36
	Disabled	2	1.81
Type of NMD	Duchenne muscular dystrophy	40	36.36
	Charcot–Marie–Tooth disease	11	10
	Becker muscular dystrophy	5	4.54
	Ullrich muscular dystrophy	1	0.9
	Spinal muscular atrophy	10	9.09
	Steiner’s myotonic dystrophy	12	10.9
	Myopathies	7	6.36
	Arthrogryposis multiplex congenita	1	0.9
	Leukodystrophy	2	1.81
	Waist muscular dystrophy	4	3.63
	Merosin-deficient-congenital muscular dystrophy	3	2.72
	Muscular dystrophy caused by a mutation in the FKRP gene	1	0.9
	Hypoplasia	1	0.9
	Spastic paresthesia	1	0.9
	Congenital myasthenia	2	1.81
	Encephalopathy	1	0.9
	Congenital muscular dystrophy with collagen VI deficiency	3	2.72
	Sarcoglycanopathy	2	1.81
	Pelizaeus-Merzbacher disease	1	0.9
	Frederich’s Ataxia	2	1.81

**Table 2:** Descriptive analysis of clinical and sociodemographic variables.

Variables	M ± SD
Parent’s age	45.15 ± 8.04
Child’s age	12.75 ± 6.56
Years of education	14.94 ± 6.91
Number of children	1.87 ± 0.97
Somatic symptomatology	12.03 ± 6.62
Dependency	47.32 ± 28.80
Overload	35.05 ± 15.50
Life satisfaction	14.39 ± 5.69
CarerQoL-Tariff	37.14 ± 17.52
CarerQoL-VAS	6.00 ± 2.19

Descriptive analyses were carried out on the psychological variables of overload, life satisfaction, somatic symptomatology, QoL, and degree of dependency (Table 2), indicating a medium level of severity in somatic symptoms, a medium level of dependency, and a mild to moderate overload. In relation to the CarerQoL, almost all informal caregivers (91%) reported at least some “satisfaction” with delivering care; 50% reported “great satisfaction”. Fifty-five percent of caregivers reported some mental health, physical or relationship problems due to caregiving. The majority of caregivers (70%) experienced problems with daily activities due to the provision of informal care. More than half of them (52%) experienced financial problems. The majority of caregivers (72%) indicated that they received support from others to carry out their caregiving tasks.

### 3.2 Second stage

In the second stage, Kruskal–Wallis analysis was conducted and those statistically significant could be seen in Table 3. All other analyses indicated no statistically significant differences. A chi-square test was conducted and those statistically significant could be seen in Table 4.

**Table 3:** Kruskal-Wallis test analysis of caregiver overload levels and clinical variables.

Variable	Caregiver overload levels	*n*	Mean rank	Mean	*H*	*p*	η^2^
Satisfaction with life	Mild	20	62.30	19.00	19.75	0.000	0.214
	Mild to moderate	37	43.82	14.64			
	Moderate to severe	29	30.12	11.62			
Somatic symptomatology	Mild	18	24.08	7.44	22.19	0.000	0.269
	Mild to moderate	35	35.83	10.82			
	Moderate to severe	25	55.74	16.76			
Psychological adaptation (social integration)	Mild	18	53.81	21.44	7.35	0.025	0.067
	Mild to moderate	33	42.55	18.57			
	Moderate to severe	32	34.80	18.25			
Psychological adaptation (self-esteem)	Mild	17	61.94	21.94	17.32	0.000	0.196
	Mild to moderate	32	36.72	16.21			
	Moderate to severe	32	34.16	16.03			
Psychological adaptation (spiritual meaning)	Mild	18	56.56	20.66	9.47	0.009	0.095
	Mild to moderate	33	38.41	14.39			
	Moderate to severe	31	36.05	13.80			
Psychological adaptation (coping efficacy)	Mild	18	54.03	20.55	7.15	0.028	0.066
	Mild to moderate	31	37.56	16.58			
	Moderate to severe	32	37.00	16.68			

**Table 4:** Chi square test of caregiver overload levels and sociodemographic variables.

	Mild	Mild to moderate	Moderate to severe	*χ* ^2^	*p*	Effect size *V*
Caregiver overload levels	*n* (%)	*n* (%)	*n* (%)			
**Marital status**				20.14	0.010	0.333
Married	19 (20.87)	30 (32.96)	17 (18.68)			
Cohabitating with a partner	1 (1.09)	2 (2.19)	3 (3.29)			
Divorced	0 (0)	3 (3.29)	5 (5.49)			
Separated	0 (0)	0 (0)	4 (4.39)			
Single	0 (0)	1 (1.09)	5 (5.49)			
**Employment**				34.86	0.004	0.438
Employed	11 (12.08)	17 (18.68)	15 (16.48)			
Unpaid work	2 (2.19)	1 (1.09)	0 (0)			
Self-employed	4 (4.39)	1 (1.09)	1 (1.09)			
Unemployed	0 (0)	8 (8.79)	6 (6.59)			
Retired	0 (0)	3 (3.29)	2 (2.19)			
Housework	3 (3.29)	7 (7.69)	2 (2.19)			
Student	0 (0)	0 (0)	6 (6.59)			
Disabled	0 (0)	0 (0)	2 (2.19)			
**Degree of dependency**				16.67	0.034	0.438
Independency	1 (1.09)	3 (3.29)	1 (1.09)			
Slight dependency	2 (2.19)	0 (0)	0 (0)			
Moderate dependency	2 (2.19)	16 (17.58)	9 (9.89)			
Severe dependency	8 (8.79)	13 (14.28)	15 (16.48)			
Total dependency	6 (6.59)	4 (4.39)	9 (9.89)			

### 3.3 Third stage

Subsequently, the variables that most explained the differences between the three groups were analyzed. For this purpose, a discriminant analysis was carried out, and the results revealed that the PHQ-15 and the SWLS are the variables that provide the strongest explanation of whether an individual will be in one overload group or another. The canonical correlation for this analysis was *r* = 0.145, with an eigenvalue of 0.022. Wilk’s lambda of the discriminant function was 0.568, χ^2^ (2, *n* = 55) = 8.815, *p* < 0.001, which indicates significant differences between the three groups.

Overall, 61% of the participants were accurately classified. The classification function accuracy was 77.9% (mild group), 42.9% (mild to moderate group), and 75% (moderate to severe group). Life satisfaction was higher and somatic symptomatology was lower in the groups that have a lower degree of overload compared to those with a moderate and severe degree of overload.

Finally, a Kruskal-Wallis test was performed to analyze the relationship between certain socio-demographic variables (occupation, marital status, sex, etc.) and somatic symptoms and satisfaction with life. Statistically significant results were obtained for caregiver occupation and somatic symptomatology (*H* = 19.42, *p* = 0.013).

### 3.4 Fourth stage

Then, a linear regression analysis was carried out. Self-esteem was a statistically significant predictor of somatic symptoms (*R*^2^ = 0.06, F (1) = 4.479, *p* = 0.038) and life satisfaction (*R*^2^ = 0.24, F (1) = 21.832, *p* = 0.000). Self-esteem explained 6% of the total variance in somatic symptoms and explained 24% of the total variance in life satisfaction. When examining the results regarding the explanatory variable of somatic symptoms and life satisfaction, self-esteem was found to be a negative predictor of somatic symptoms (*β* = −0.29, *t* = −2.11, *p* = 0.038) and a positive predictor of life satisfaction (*β* = 0.49, *t* = 4.67, *p* = 0.000).

### 3.5 Fifth stage

Finally, an analysis was conducted to determine whether certain sociodemographic variables were associated with the last mentioned variable: self-esteem. The only variable for which statistically significant differences were found was caregiver occupation for self-esteem (*H* = 19.04, *p* = 0.015).

**Figure 2:**
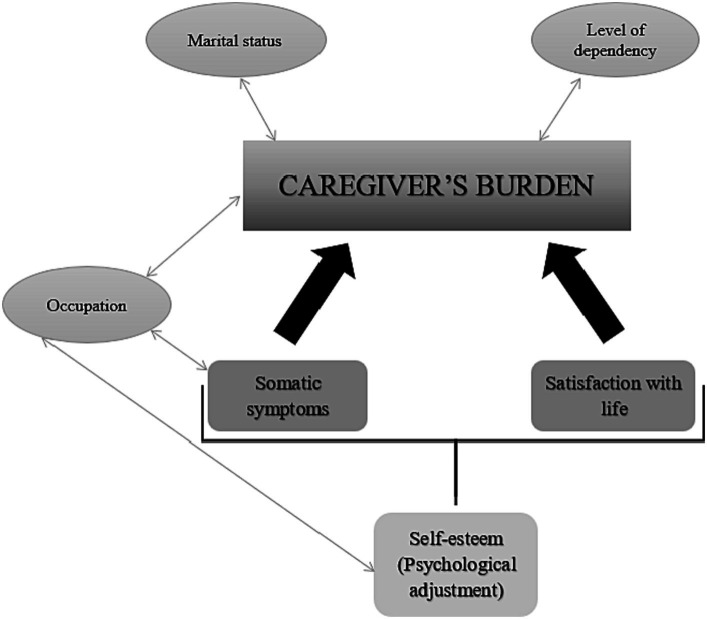
Variables that influence caregiver’s overload.

The overall model is represented in Figure 2.

## 4 Discussion

The present study identified a number of associated variables for caregiver overload in parents of children with NMDs. Variables such as marital status and child’s degree of disability influence overload, and occupation influences this variable and somatic symptoms and self-esteem. It was observed that the presence of somatic symptomatology and life satisfaction predicted that the caregiver might suffer a degree of overload. Similarly, the caregiver’s self-esteem when facing the child’s illness was found to influence somatic symptomatology and life satisfaction.

These results highlight the most important finding of this research. The effects of caregiving on the caregiver’s physical and psychological status are crucial in determining their degree of overload, as well as their sociodemographic status.

### 4.1 Descriptive analysis

Several psychological variables were analyzed. Caregivers suffer from somatic symptomatology with a medium level of severity. This is consistent with other studies in this population, which stated that physical problems may develop due to prolonged care (4). These results were similar to those of other studies with informal caregivers of people with other pathologies (57, 58). An explanation for this was given by Gräsel (59), who suggested that caregiving tasks such as lifting the patient out of bed might explain limb disorders and consequently skeletal system complaints.

Regarding satisfaction with life, due to the differences in scale adaptations, comparisons are only possible within the Spanish population. However, we did not find any studies that were carried out among informal caregivers. In addition, no international studies were found in which outcomes were analyzed with z scores. The mean life satisfaction score in the present study was lower than the mean score in other studies conducted with “normative” population data (60–62). Parents of children with NMDs will have to live with a disease throughout life and that it has probably taken them years to get a diagnosis. According to several studies, the event that produces the highest levels of stress and upset is the time of diagnosis (4, 27). When children have been diagnosed, they have found that there is no curative treatment, which is frustrating for many families. In addition, many of these NMDs are progressive and very disabling, resulting in the patient being bedridden, with the caregiver spending most of the day looking after the patient and accompanying them in daily activities. This is added to feelings of uncertainty as to whether the situation will worsen or the fear of losing their loved one in the near future.

As a result, it is not surprising then that, in the CarerQoL, the majority of caregivers reported mental health, physical, social relationship, and financial problems as a consequence of caring for children with NMDs. Compared to other studies, such as de Kanters et al. (63) (who studied Pompe disease) and Pangalila et al. (64) (who studied Duchenne muscular dystrophy), parents in the present study had more problems in activities of daily living, more physical problems and emotional distress. However, it was also found that almost all of them reported at least some “satisfaction” in providing care and that they received support from others in carrying out their caregiving tasks since the care experience can sometimes be satisfying.

Additionally, the caregivers’ overall QoL scores (CarerQoL-Tariff) in the present study were lower than the scores reported by other studies with informal caregivers of children with other diseases (51, 65) and studies with informal caregivers of adults with chronic conditions (53).

The results also showed that parents had a higher level of overload than informal caregivers in other studies with the same population (33, 66) and with chronic diseases (67, 68). These levels of overload were explained by certain sociodemographic variables.

### 4.2 Sociodemographic variables related to overload

The present study found that occupation, marital status and the child’s level of dependency were related to caregiver overload. Caregivers who were not married or had no partner, had no job, or had a child with a severe level of dependency were at higher levels of caregiver overload. In general, the level of dependency is directly related to a higher number of hours spent on caregiving tasks. This can result in caregivers losing their job or taking more days of sick leave, reducing their leisure time and social life, and lack of personal fulfillment (69). It has been seen that unemployment status and, consequently, low-income levels have been a significant barrier to the majority of caregivers (70).

Relatedly, being single or separated has been shown to be associated with a higher overload. Most of caregivers do not stop working, which does not allow them to combine their work with their caring duties and limits their leisure time. The greater overload can also be explained by the household income, which is lower because there is only one salary (71).

### 4.3 Predictors of overload

One of the most relevant findings of the present study is the identification of 3 overload groups based on life satisfaction and somatic symptom scores within the predictive model of the discriminate analysis. In the literature, somatic symptoms have been associated with the likelihood of caregiving stress and mood disorders (72). This relationship is explained by Pagnini et al. (66), who state that somatic symptoms precede the perception of overload. Against this hypothesis proposed by these authors, other authors found that within these somatic symptoms, fatigue and sleep disturbances have been associated with increased caregiver overload (57). Regarding life satisfaction and overload, the mechanisms underlying this relationship may be that life satisfaction is produced by the positive aspects of caregiving (73). If informal caregivers find positive aspects of caregiving, they may improve their life satisfaction and not suffer from overload despite having many caregiving tasks (31, 74). However, positive aspects of caregiving cannot be shown in this study to be a mediating variable, as they were not measured.

Positive aspects of caregiving can lead to a higher perception of reward and increased self-esteem (73). These aspects may be a protective factor since a positive caregiving experience has been reported to result in good QoL in caregivers who were severely overburdened. Positive aspects increase as the caregiver’s role is inspiring and rewarding, enriching their life experiences. Positive aspects in the literature typically include satisfaction, rewards, competence, benefit, meaning, personal growth and sense of duty (75).

Likewise, the variable self-esteem was an explanatory variable for predictors of overload (namely somatic symptomatology and life satisfaction). This relationship is consistent with other studies conducted with RD caregivers and other conditions, which found a relationship between self-esteem and life satisfaction (76) and somatic symptoms (57). High self-esteem consists of an individual respecting himself and considering himself worthy (77). In the present study, it refers to the perceived ability to cope with challenges and threats (23). This factor is included within the psychological adaptation to the disease and constitutes a protective factor against the diagnosis of the disease, indicating higher resilience (23).

The caregiver’s occupation also influenced having higher somatic symptomatology and lower self-esteem. Caregivers who were unemployed had a lower level of self-esteem and higher somatic symptomatology, which is consistent with other studies (78). The importance of this variable should be considered, since unemployment leads to difficulty in addressing the child’s needs (access to medical resources or technical aids). It is a major source of stress for the family, leading to a lower perception of the condition’s management, which may cause feelings of helplessness and hopelessness, resulting in physical and mental problems, increasing somatic symptoms (79) and reducing self-esteem (80). These results were also found in a study conducted among parents of children with dystrophinopathies and suggest the need for government-supported out-of-home day care or respite care for children with DMD, thus allowing parents to escape, even temporarily, from the stress and overload of caring for these children. This is because employment can provide respite from the demands of childcare.

The predictive variables of overload in the present study are congruent with other studies conducted with caregivers of chronically ill people, which conclude that overload is influenced by physical and psychological health, self-perception (self-esteem), the child’s functional ability and caregiving demands (14, 15).

In sum, caregivers of children with NMDs face daily caregiving challenges that cause a decline in their physical and psychological health. It is essential to know which factors may favor the appearance of these problems to prevent them and intervene appropriately. Specifically, providing external support such as assistance from formal caregivers could prevent physical problems (contractures, back problems, muscle pain) associated with caregiving tasks. In addition, financial support for financially vulnerable families can reduce physical and psychological problems. Thus, these results provide guidelines for implementing new support programs for these families to meet their emotional and socio-economic needs. Due to the increase of somatic symptoms, organisations and governments should provide physiotherapy sessions in order to alleviate these symptoms, as well as psychological support programs using cognitive-behavioral techniques for the reduction of negative and irrational thoughts related to their child’s illness and assertiveness training. In this way, they would be working to improve their life satisfaction and self-esteem. In addition, and due to the unemployment rate, a suitable work reintegration program, as well as the availability of financial support for caregiving, could reduce the overload.

### 4.4 Limitations and future lines of research

The study has certain limitations that should be acknowledged. These include the extended duration and complexity of the research protocol. Additionally, challenges arose when attempting to draw comparisons among various international adaptations of the SWLS, as previously noted. Furthermore, the PAS instrument did not provide an exhaustive measure of the diversity of coping strategies, so it was not possible to go into these aspects in more detail. Moreover, the convenience sampling method employed might be considered a limitation, as the rarity of RDs made it impractical to randomly select participants. Another potential limitation is the exclusion of other family caregivers, such as siblings, uncles, aunts, and grandparents, which could have impacted the study’s comprehensiveness. Lastly, despite encompassing a range of neuromuscular diagnoses, the sample size lacked balance. Despite the diversity of diagnoses, all had similar symptoms, which are presented in the introduction. On the other hand, the sample was predominantly married or partnered females, which limits the generalizability of results to the caregiver population. In addition, the employment status of the partners was not asked, which is a relevant variable that may influence overload and should be considered in future studies.

Future lines of research could aim to produce a standard protocol to be able to consistently assess the physical and psychological status of caregivers of patients with neuromuscular pathologies to carry out interventions that consider the specific needs of this group. On the other hand, other non-parental caregiver perspectives, such as siblings, grandparents or aunts and uncles, could be included in future research. Moreover, a longitudinal study would be appropriate to assess how the overload experienced by caregivers of children with NMDs evolves over time. Future studies could include other rare diseases, such as motor or endocrinological diseases, and include new variables that have been shown to influence overload in other studies, such as social support or children’s behavioral problems.

### 4.5 Implications for practice

The practical implications of the present study indicate that a good occupation, good self-esteem and life satisfaction, and the absence of somatic symptomatology can protect caregivers against overload. Interventions for families of children with NMD should therefore consider these factors, as well as those related to the health and social systems. Therefore, a family-centered approach that recognizes the family as central to the child’s health may be helpful by including comprehensive support not only for the child diagnosed with NMD but also for the family, for example, with participation in a parent-to-parent support group (81). This is because parents who are supportive, involved and have positive attitudes have a higher level of resilience with respect to stress induced by their children (10).

## 5 Conclusion

This study has identified factors associated with overload in parents of children with NMDs, and some of them were predictors. Being unmarried or having no partner, having no job, and having a child with a severe level of dependency is associated with an increase the levels of caregiver overload. It was observed that the presence of somatic symptomatology and life satisfaction predict that the parent may suffer some level of overload. Likewise, the caregiver’s self-esteem with respect to the child’s illness can influence somatic symptomatology and life satisfaction. Finally, unemployment is a major risk factor for health problems induced by caregiving.

Working on reducing caregivers’ somatic symptoms, avoiding physical problems, and improving their life satisfaction and self-esteem could help to reduce the problems caused by the impact of the disease on the caregiver. Knowing the predictors of caregiver overload is important for professionals who are in charge of developing psychological support programs. This analysis emphasizes the importance of paying special attention to the presence of certain symptoms in caregivers and paying attention to a person’s environment to prevent overload.

Finally, the analysis of the relationship between sociodemographic and psychosocial predictors is essential; this can be helpful when reviewing the current protocols, assessing dependency, and highlighting the areas with the greatest impact on the caregiver. Therefore, the experience of caregiving does not have to be detrimental to one’s physical and psychological health.

## Data availability statement

The raw data supporting the conclusions of this article will be made available by the authors, without undue reservation.

## Ethics statement

The studies involving humans were approved by the ethics committee of the University of Deusto (Ref: ETK-39/18-19). The studies were conducted in accordance with the local legislation and institutional requirements. The participants provided their written informed consent to participate in this study.

## Author contributions

AR: Data curation, Methodology, Writing – original draft, Writing – review & editing. MG: Conceptualization, Investigation, Writing – review & editing. OM: Formal analysis, Methodology, Validation, Writing – original draft. JL-P: Methodology, Supervision, Writing – review & editing. IG: Conceptualization, Investigation, Writing – original draft. PP-N: Conceptualization, Methodology, Supervision, Writing – review & editing. IA: Writing – review & editing, Writing – original draft.
